# TeenCovidLife: a resource to understand the impact of the COVID-19 pandemic on adolescents in Scotland

**DOI:** 10.12688/wellcomeopenres.17252.1

**Published:** 2021-10-18

**Authors:** Charlotte F Huggins, Chloe Fawns-Ritchie, Drew M Altschul, Archie Campbell, Clifford Nangle, Rebecca Dawson, Rachel Edwards, Robin Flaig, Louise Hartley, Christie Levein, Daniel L McCartney, Stephanie L Sinclair, Clare Dolan, Dawn Haughton, Judith Mabelis, Judith Brown, Jo Inchley, Daniel J Smith, Ian J Deary, Caroline Hayward, Riccardo E Marioni, Andrew M McIntosh, Cathie Sudlow, David J Porteous

**Affiliations:** 1 Centre for Genomic and Experimental Medicine, Institute of Genetics and Cancer, University of Edinburgh, Edinburgh, UK; 2 Department of Psychology, University of Edinburgh, Edinburgh, UK; 3 Centre for Medical Informatics, Usher Institute, University of Edinburgh, Edinburgh, UK; 4 MRC Human Genetics Unit, Institute of Genetics and Cancer, University of Edinburgh, Edinburgh, UK; 5 Centre for Biomedicines, Self and Society, Usher Institute, University of Edinburgh, Edinburgh, UK; 6 MRC/CSO Social and Public Health Sciences Unit, University of Glasgow, Glasgow, UK; 7 Division of Psychiatry, Royal Edinburgh Hospital, University of Edinburgh, Edinburgh, UK

**Keywords:** adolescence, COVID-19, mental health, longitudinal study, observational study, lockdown, well-being

## Abstract

TeenCovidLife is part of Generation Scotland’s CovidLife projects, a set of longitudinal observational studies designed to assess the psychosocial and health impacts of the COVID-19 pandemic. TeenCovidLife focused on how adolescents in Scotland were coping during the pandemic. As of September 2021, Generation Scotland had conducted three TeenCovidLife surveys. Participants from previous surveys were invited to participate in the next, meaning the age ranges shifted over time.

TeenCovidLife Survey 1 consists of data from 5,543 young people age 12 to 17, collected from 22 May to 5 July 2020, during the first school closures period in Scotland. TeenCovidLife Survey 2 consists of data from 2,245 young people aged 12 to 18, collected from 18 August to 14 October 2020, when the initial lockdown measures were beginning to ease, and schools reopened in Scotland. TeenCovidLife Survey 3 consists of data from 597 young people age 12 to 19, collected from 12 May to 27 June 2021, a year after the first survey, after the schools returned following the second lockdown in 2021. A total of 316 participants took part in all three surveys.

TeenCovidLife collected data on general health and well-being, as well as topics specific to COVID-19, such as adherence to COVID-19 health guidance, feelings about school closures, and the impact of exam cancellations.

Limited work has examined the impact of the COVID-19 pandemic on young people. TeenCovidLife provides relevant and timely data to assess the impact of the pandemic on young people in Scotland. The dataset is available under authorised access from Generation Scotland; see the Generation Scotland website for more information.

## Introduction

The coronavirus disease 2019 (COVID-19) pandemic has affected the lives of people of all ages across the world. In the UK there have now been two national lockdowns, in which schools and workplaces were closed and non-essential travel was stopped. Moreover, the general population has been asked to make ongoing changes to their lifestyle to minimise the risk of contracting and transmitting the disease. This upheaval to everyday life may have long-term socioeconomic and psychological effects, necessitating careful documentation and study ^1^.

This paper describes the TeenCovidLife dataset, a dataset collected by Generation Scotland on the health and well-being of adolescents in Scotland. This dataset is available through authorised access in the UK and abroad for use in research. More details on the Generation Scotland access procedure can be found on the Generation Scotland website.

Generation Scotland is a long-running family and population-based health study. Since 2006, Generation Scotland has been gathering data and collaborating with researchers to produce high-quality health research across many fields ^2^. Moreover, longitudinal population studies such as Generation Scotland are particularly well-positioned to study the COVID-19 pandemic ^3^. This led to the formulation of the CovidLife project, studying the impact of COVID-19 on over 18,000 adults in the UK ^4^.

Findings from the CovidLife and other longitudinal population studies revealed that young adults showed elevated risks of depression and anxiety during the pandemic ^5^. Likewise, Chinese adolescents during the early stages of the pandemic showed higher than usual levels of depression and anxiety ^6^. This suggests young people’s mental well-being may be negatively impacted by the pandemic. Despite this, there is still little cohort research capturing adolescent’s experiences of the COVID-19 pandemic.

The TeenCovidLife project was designed to address this important gap in the research literature. In this series of three surveys, over 7,000 young people age 12 to 19, living in Scotland, completed questionnaires about their experiences, feelings, and well-being during the COVID-19 pandemic. The first survey was conducted from May to July 2020, during the first pandemic-related school closures in Scotland. The second survey was conducted from August to October 2020, when lockdown measures were being relaxed and schools reopened. The third survey was conducted approximately one year after the first, from May to June 2021, when most schools had reopened after the second national lockdown.

## Methods

### Materials & Methods

***Questionnaire Development.*** The TeenCovidLife questionnaires were developed using Qualtrics survey software, a survey development tool ^7^, with versions dated May 2020, August 2020 and May 2021 for each wave of the survey. Data collection was limited to remote online assessments due to the COVID-19 restrictions. However, this also enabled quick data capture, allowing the sampling of psychological and health data at different stages of the pandemic. The online survey was suitable for completion across many devices, including desktop computers, tablets, and smartphones. The surveys were developed and tested by the Generation Scotland team at the University of Edinburgh, in collaboration with the Schools of Health and Wellbeing Improvement Research Network ( SHINE) based at the University of Glasgow.

Given the sensitivity of some of the questions, as well as potential reservations about providing personal information in an online study, none of the questions in the surveys required an answer. Many sensitive questions also had a “prefer not to answer” option. If participants left a question unanswered, they were asked to confirm if they wanted to continue without answering. For data privacy reasons, after moving to the next page of questions, participants were not able to go back and amend their answers. As Qualtrics does not have password-protected accounts, his was to prevent other people in the same household from using the same device to view the participant’s responses.

The questions included in all three TeenCovidLife surveys can be seen in the *Extended data* ^8^. A copy of the Qualtrics survey (.qsf) for any survey can also be requested from the authors.

***Measures.*** TeenCovidLife assessed general well-being and young people’s experience of the pandemic. The topics assessed across the three surveys include:

A. Education and employment

■ Ability to adapt to home learning
■ Worries about studies
■ Impact of the cancelled exams
■ Satisfaction with 2020 exam grades
■ Preference for face-to-face compared to online learning
■ Impact on further education and current/future employment

B. COVID-19 knowledge and health behaviours

■ Knowledge of COVID-19
■ Understanding of, and trust in, health guidance
■ Adherence to, and support of, COVID-19 health guidance
■ Vaccine opinions and hesitancy

C. Well-being and mental health

■ Loneliness and social support
■ Well-being and life satisfaction
■ Worry about COVID-19 and impact on future
■ Sleep quality and social media use

Measures were selected to harmonise both with other studies and work by the Wellcome Longitudinal Population Studies secretariat, as well as on-going Generation Scotland and SHINE work, including the Health Behaviour in School-Aged Children (HBSC) study ^9^ and the SHINE online pupil mental health survey. Using similar items to other studies enables replication and further collaboration with other population health studies. Novel questions were also created to assess responses specific to COVID-19, as few well-validated measures about COVID-19 existed at the time.

Some questions only appeared in one survey. If a participant had taken part in previous surveys, not all questions were asked again as some items were judged as unlikely to have changed between surveys.

Several commonly used psychological measures were presented in all three surveys:

- The Adolescent Sleep-Wake Scale (ASWS) ^10^, a ten-item measure assessing sleep quality and disturbances in adolescents.
- Brief Resilience Scale (BRS) ^11^, a short measure assessing trait resilience – the ability to “bounce back” from setbacks and distress.
- Perceived Stress Scale (PSS-4) ^12^, a four-item measure assessing current stress.
- World Health Organisation Well-Being Index (WHO-5) ^13^, a five-item measure assessing overall wellbeing.

Several subscales of the Social Emotional Health Survey (SEHS) ^14^ were also applied, assessing the level of social support from family members, friends, and school staff, as well as optimism and self-efficacy. See Table 1 for further details of measures used in all surveys, as well as the *Extended data* ^8^ for full questionnaires.

**Table 1.** Details of measures used in the TeenCovidLife surveys.

	Outcome	# Qs	Source	Version	Repeated	Asked to
**Demographics**						
Age	-	1	-	All	*	All
Sex	-	1	-	All		All
Gender Identity	Whether participant’s gender differed from their sex as assigned at birth If so, collected gender identity information.	2	-	All		All
Ethnic Origin	-	1	-	All		All
Medical Condition	If participant had long-term health condition, such as asthma or diabetes.	1	-	All		All
Carer Status	Indicated household members, if any, the participant had caring responsibilities for	1	-	All		All
Postcode	-	1	-	TCL1	-	All
Autism Status	If participant had a diagnosed Autism Spectrum Condition	1	-	TCL2	-	All
ADHD Status	If participant had diagnosed Attention Deficit Hyperactivity Disorder	1	-	TCL2	-	All
**General Education**						
Pupil Status	If participant was a secondary school pupil	1	-	All	*	All
School Year	-	1	-	All	*	School Pupils
Feelings on School	How participants felt about school and how pressured they felt by schoolwork.	2	HBSC Scotland ^9^	All	*	School Pupils
School Location	Where participants were doing school work (e.g., at home or still attending school), and how difficult they found changing to do schoolwork at home	2	-	TCL1	-	School Pupils
School Resources	If participants had an appropriate device and physical space in which to do their schoolwork	2	-	TCL1	-	School Pupils
Plans after school	If participants left school in Spring 2020, and if so what they planned to do afterwards, and if these plans had changed due to Covid-19	3	-	TCL2+3	-	Age 16+
School Bullying	How frequently participant was bullied by other young people	1	-	TCL2+3	-	School Pupils
**Impact of Covid-19 on School**						
Worry about returning to school	How much participant worried about returning to school after the first national lockdown	1	-	TCL2+3	-	School Pupils
Safety in returning to school	How much participant agreed that it was safe to return to school as the lockdown measures eased	1	-	TCL2+3	-	School Pupils
Looking forward to school	-	1	-	TCL2	-	School Pupils
Missing aspects of school	How much participants missed seeing friends and teachers from school	2	-	TCL2	-	School Pupils
Worry about studies	How much participant worried about their grades and being on track with their studies	2	-	TCL2+3	-	School Pupils
Worry about school-based COVID transmission	How much participant was worried that returning to school would increase their own, their family’s or their teacher’s chance of contracting Covid-19	2	-	TCL2+3	-	School Pupils
Online vs face-to-face schooling	Whether participants preferred remote, online, or hybrid learning, why, and if online or face-to-face schooling was worse.	4	Common Sense Media ^15^	TCL3	-	School Pupils
Technology access	Whether participants had access to technology to learn remotely and if they had been issued a device in any national initiatives	2	-	TCL3	-	School Pupils
School challenges	What participant’s biggest challenges for schoolwork were over the past year	1	Common Sense Media ^15^	TCL3	-	School Pupils
In-School Testing	If participant is taking part in school testing, and motivations for taking part/not taking part	6	-	TCL3	-	School Pupils
**Exams**						
Exams	How many and what types of exams pupils were expecting to sit in 2020	1	-	TCL1	-	School Pupils
SQA Results	If participant received SQA results in 2020	1	-	TCL2	-	All
Grades Fairness	How fair participants viewed the different methods used to estimate SQA grades	4		TCL2	-	Received SQA Results
Grades Changed	If the participant had their initial SQA results changed in August 2020	1	-	TCL2	-	Received SQA Results
Grade Satisfaction	How happy participants felt about their final grades	1	-	TCL2	-	Received SQA Results
Exam Comparison	Extent to which participants feel their grades would have been better or worse had they sat exams in Spring 2020	1	-	TCL2	-	Received SQA Results
SQA Results Worry	How worried participants felt about the impact their SQA results would have on their employment and education in future	2	-	TCL2+3	-	Received SQA Results
Exam Cancellation Worry	How worried participants felt about the impact of exam cancellations on their own and others grades	2	-	TCL3	-	School Year > S3
**Employment**						
Job Before Lockdown	If participant was employed **before** the first lockdown in 2020	1	-	All		Age 16+
Changes to employment	If participant had lost job, was furloughed or experienced a pay cut due to Covid-19	1	-	All	*	Employed
Key Worker Status	If participant had been designated a key worker	1	-	All	*	Employed
Job Now	If participant was employed at time of survey	1	-	All	*	Age 16+
Working Hours	-	1	-	TCL1+2	*	Employed
PPE at Work	If participants job required them to have close contact with others, and, if so, how frequently they had appropriate personal protective equipment	2	-	TCL2+3	-	Employed
**Household Factors**						
Accommodation Type	Type of home the participant lives in	1	-	All		All
Household Size	Number of people participant lives with and who these people were in relation to participant	2	-	All		All
Rooms in House	-	1	-	All		All
High Risk	If anyone in participant’s household had received shielding letter	1	-	TCL1	-	All
Leaving Household	How frequently the participant saw people outside of their household, and who these people were in relation to the participant	1	-	TCL1	-	All
Garden	If participant had access to a garden or yard	1	-	TCL1	-	All
Pet	If participant had any pets, and if so what kind.	2	-	TCL1+3	-	All
Impact of Pet	Impact pet had on coping, family and fitness during pandemic	4	Ratschen, Shoesmith ^16^	TCL3	-	Pet owners
Parent Key Worker	If participant’s parent was designated as a key worker or not	1	-	TCL1	-	All
Parent Work Situation	If parents were working or not, and whether they were working from home	3	-	TCL1	-	All
Digital Access	What digital resources participant had access to (e.g., smartphone, landline, desktop, etc).	1	-	TCL1	-	All
**Covid-19 Factors**						
Covid-19 Infection	If participant had suspected or confirmed Covid-19 infection	1	-	All	*	All
Household Covid-19 infection	If someone in participant’s household had suspected or confirmed Covid-19 infection	1	-	All	*	All
Public Health Threat	Extent to which participants believed Covid-19 constituted a public health threat	1	-	All	*	Age 15+
Covid-19 Knowledge	How knowledgeable participants feels about Covid-19	1	-	TCL1	-	Age 15+
Understanding of Guidance	How easy the participant found understanding health guidance around Covid-19	1	-	TCL1	-	Age 15+
Time Learning about Covid-19	How long the participant felt they spent getting news about Covid-19 each day	1	-	TCL1	-	Age 15+
Impact on Routine	Degree to which Covid-19 impacted participant’s routine	1	-	TCL1	-	All
Life Impact	How positively or negatively theCovid-19 pandemic impacted participants’ lives	1	-	TCL2+3	-	All
Covid-19 Guidance	How easy participant found Scottish and UK Government guidance to understand	2	-	TCL2+3	-	Age 15+
Trust in Medical Advice	How much participant trusted medical advice from the UK Government, the Scottish Government, and from medical workers	3	-	TCL2	-	Age 15+
Covid-19 Mitigation Behaviours	How frequently participants were washing their hands, wearing face coverings in enclosed spaces and keeping distance from people outside the household	3	-	TCL2+3	-	All
Covid-19 Mitigation Motivations	Motivations for maintaining social distancing	1	Oosterhoff, Palmer ^17^	TCL3	-	All
Isolation Behaviour	How likely participant would be to self-isolate if they had come into contact with a positive Covid-19 case	1	-	TCL2+3	-	All
Face Covering Support	If participant agrees or disagrees about whether people should wear face coverings in enclosed spaces	1	-	TCL2+3	-	All
**Vaccines**						
Vaccine Attitudes	How much participants agree or disagree that vaccines were safe, effective and important	3	Wellcome Global Monitor ^18^	TCL2	-	All
Vaccine Uptake	Degree to which participant though they would want to be vaccinated, and whether their parent would want them to be vaccinated.	2	Wellcome Trust LPS Questionnaire ^19^	TCL2	-	All
Vaccine Worry	How worried participants were about under 16s not being included on the vaccine roll-out plans	1	-	TCL3	-	All
**Mental Health & Well-Being Outcomes**						
WHO-5	*World Health Organisation 5 – Well-being Index.* A measure of global well-being	5	WHO ^13^	All	*	All
PSS-4	*Perceived Stress Scale 4* – measure of self- reported stress.	4	Cohen, Kamarck ^12^	All	*	All
BRS	*Brief Resilience Scale* – measure of resilience and ability to ‘bounce back’	6	Smith, Dalen ^11^	All		All
SEHS	*Socio-Emotional Health Survey* –measure of social and emotional well-being in children across 5 subscales: Optimism, Self-Efficacy, Family Support, Peer Support, Support at Home and Support at School.	15	Furlong, You ^14^	All	~ ^1^	All
Good Childhood Index	Measure of general life satisfaction in children across 5 domains: school, the future, friendships, home, family, and life.	6	The Children's Society ^20^	All	* ^2^	All
ASWS	*Adolescent Sleep Wake Scale (10 Item)* measure of general sleep quality in adolescents.	10	10	All	*	All
Sleep Quality	Sleep quality compared to previous time point, as well as change in bed times.	4	-	TCL1+2	*	All
General Health	Self-perceived general health	1	36-item Short Form Survey ^21^	All	*	All
Current Loneliness	Frequency of loneliness over past week	1	-	All	*	All
Pre-Pandemic Loneliness	How lonely participant felt **before** the first Covid-19 lockdown	1	-	TCL1	-	All
Future Worry	Degree of worry about the future	1	-	All	*	All
Job Worry	Degree of worry about losing job	1	-	All	*	Employed
Hobby Worry	Degree of worry about time for hobbies	1	-	TCL1+2	*	All
Worry Education	Degree of worry participant felt about Covid- 19’s impact on their exams and education	2	-	TCL1	-	All ^3^
Worry Work Experience	Degree of worry participant felt about Covid- 19’s impact on their work experience	1	-	TCL1	-	15+
Worry Family Life	Degree of worry participant felt about arguing with members of their family, or family members arguing with one another	1	-	TCL1	-	All
Worry Contact	Degree of worry participant felt about their ability to see friends and family	2	-	TCL1	-	All
Happiness Comparison	How happy participant felt they were this time last year	1	-	TCL2+3	-	All
**Leisure**						
Social Media Use	How much time participants felt they spent looking at and using social media compared to previous time point	1	-	All	*	All
New Skills & Hobbies	What hobbies participants had taken up over past year of the pandemic	1	CovidLife ^4^	TCL3		All

*Notes.* - in *Source* indicates that question was formulated in-house. * indicates that question was asked to previous participants again in TCL2 and TCL3. ^1^ Not all domains of the SEHSS were repeated. For participants who took part in a previous survey, they were only asked about Optimism and School Support at TCL2 and TCL3. Only mentioned subscales of the SEHSS was used; this is not the full questionnaire. ^2^ Not all domains of the Good Childhood Index were used in TCL2 and TCL3. ‘Satisfaction about Home Life’ was not included in TCL2+3. ^3^ Question about education was asked to all, question about exam only asked to participants who were expecting to sit an exam in 2020

### Sample

***TeenCovidLife Survey 1.*** Anyone who was aged 12 to 17 and resident in Scotland was able to take part in the study. As this was an online survey, internet access was required to participate. The questionnaire could be accessed using any device, including a tablet or smartphone. Data collection commenced on Friday 22 ^nd ^May 2020 and closed Sunday 5 ^th^ July 2020, during the first coronavirus-related school closure period in Scotland, which lasted from 23 ^rd^ March to 11 ^th^ August 2020. The recruitment period lasted a total of 44 days.

**Figure 1.**
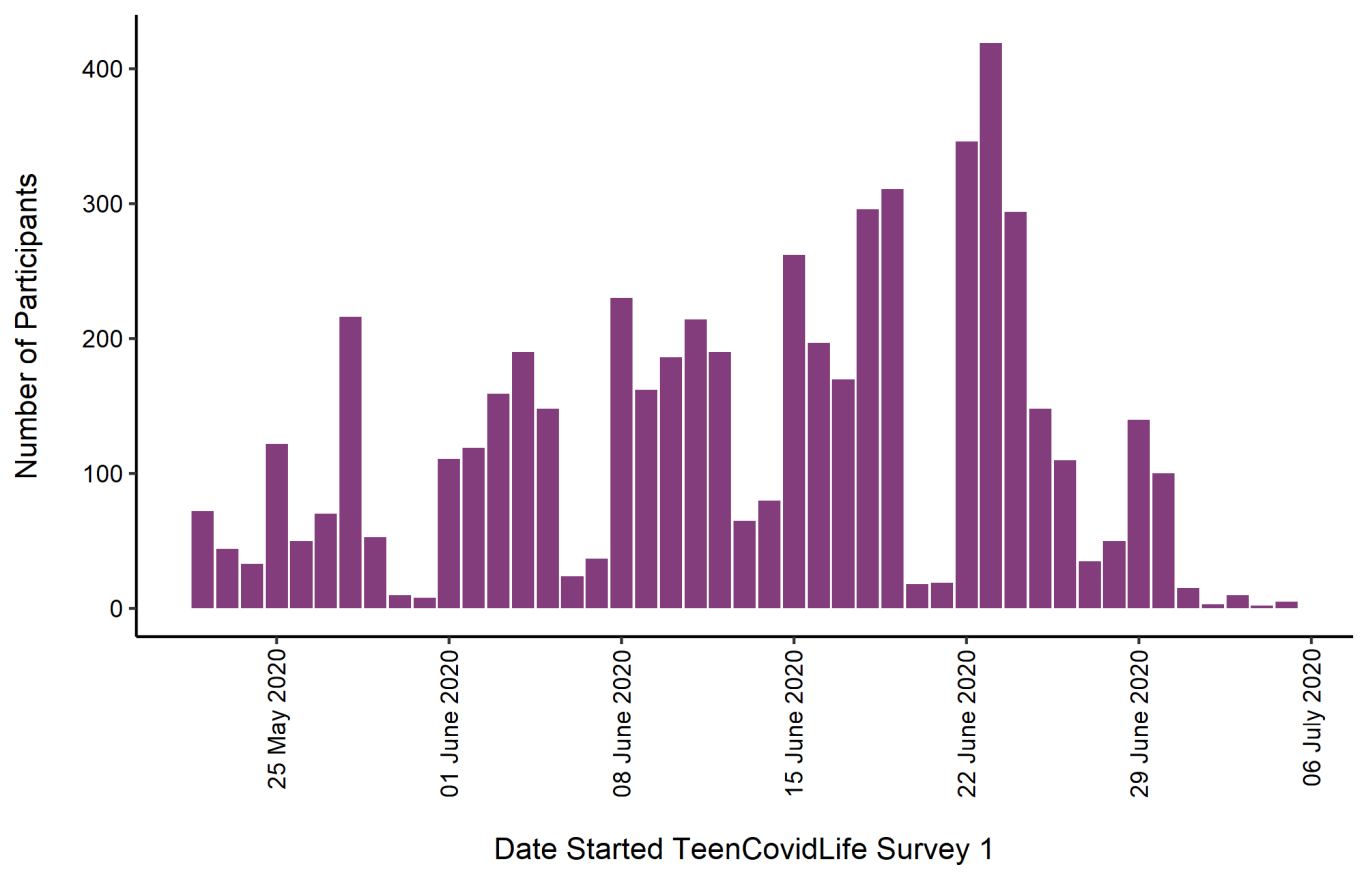
Sample of TeenCovidLife Survey 1 by date they started the survey.

After participants began the survey, they had seven days to complete it. The final sample consisted of 5,543 young people age 12 to 17. Figure 1 shows the number of participants included in the final sample for Survey 1 by the day they began the survey.

***TeenCovidLife survey 2.*** All participants in TeenCovidLife Survey 1 with a working email, who consented to re-contact were sent an email invite. This included any participants who may have turned 18 since the first questionnaire. As such, the potential age range for returning participants was 12 to 18. However, a separate Qualtrics survey was set up for any participants who had not taken part in TeenCovidLife Survey 1. These participants needed to be age 12 to 17 and living in Scotland. The ‘New’ and ‘Repeat’ versions of the surveys only differed in that some items (such as sex) were not asked again to previous participants. The full questionnaires can be seen in the *Extended data* ^8^. As before, internet access was required to take part.

Data collection took place from Tuesday 18 ^th^ August 2020 to Saturday 10 ^th^ October 2020, closely following the initial re-opening of schools on the 11 ^th^ of August. The recruitment period lasted 54 days. After participants began the survey, they had 14 days to complete it.

**Figure 2.**
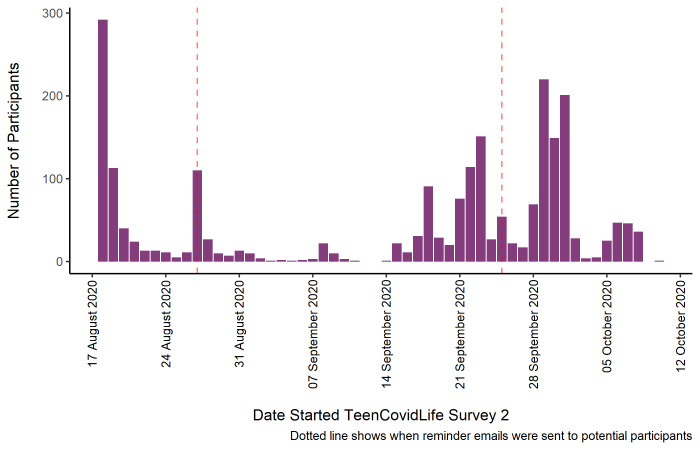
Sample of TeenCovidLife Survey 2 by date they started the survey.

The final sample consisted of 2,232 young people age 12 to 18. Of this sample, 761 had taken part in TeenCovidLife Survey 1. See Figure 2 for the numbers included in the final sample for Survey 2 by the day they began the survey, as well as when reminder emails were sent to previous participants.

***TeenCovidLife Survey 3.*** All participants who took part in either previous TeenCovidLife surveys and gave permission for re-contact along with a working email address were invited to take part. Some returning participants may have turned 18 or 19 since the first survey. Consequently, the returning sample ranged between ages 12 to 19.

As before, young people age 12 to 17 living in Scotland who had not taken part in a previous survey were also able to take part. As in Survey 2, two Qualtrics surveys were created for new and repeat participants. These only differed in that some items, such as sex, were not asked again to repeat participants. Both questionnaires for Survey 3 can be seen in the *Extended data* ^8^.

Data collection began Tuesday 12 ^th^ May 2021 and ran until Sunday 27 ^th^ June 2021. Data collection took place when students were returning to school after another period of school closures. The end-date for data collection was chosen as this was when most schools in Scotland closed for the summer holidays. The recruitment period lasted a total of 46 days. After participants began the survey, they had 14 days to complete it.

**Figure 3.**
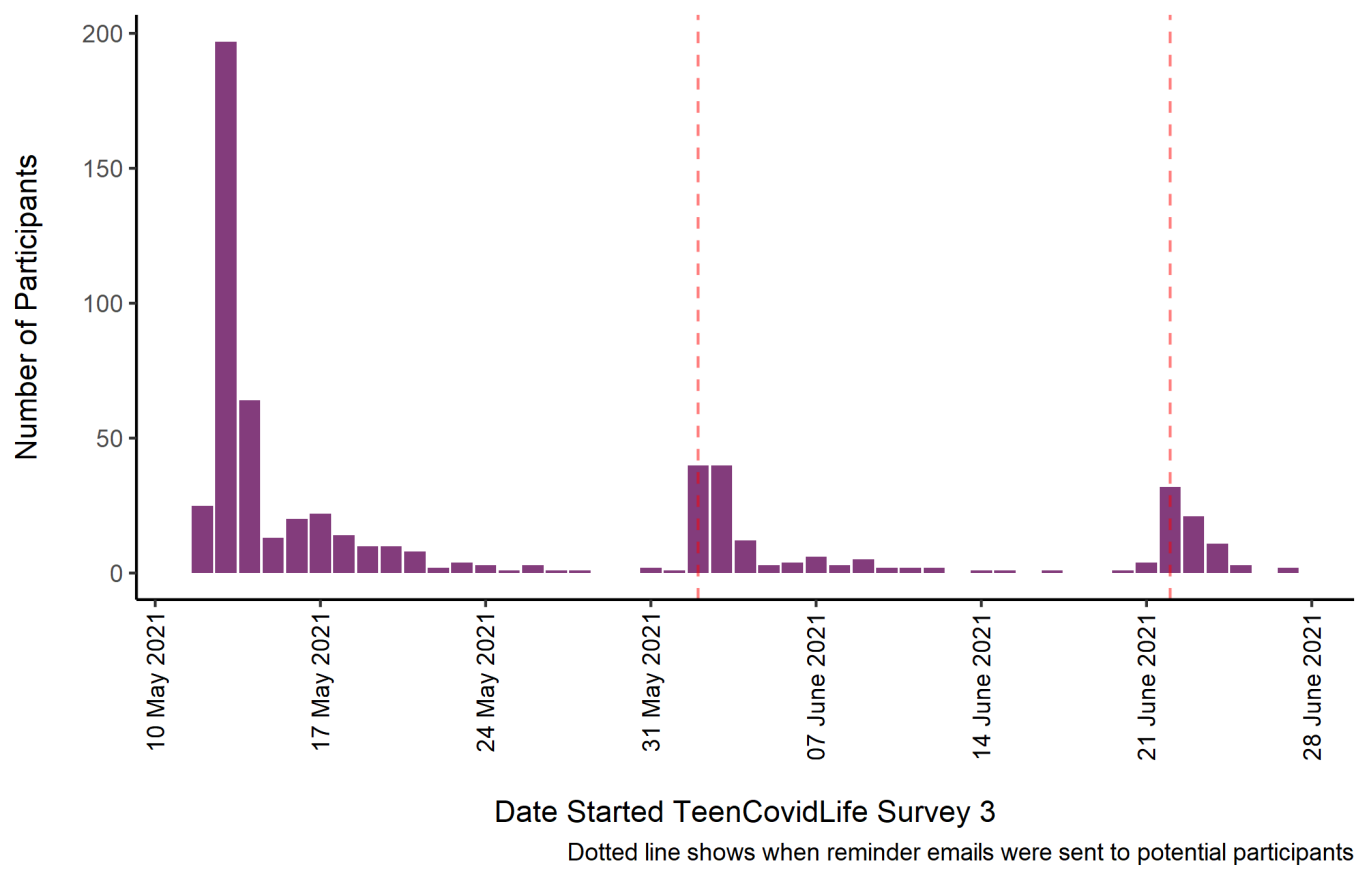
Sample of TeenCovidLife Survey 3 by date they started the survey.

The final sample consisted of 597 young people age 12 to 19 years old. Of the full Survey 3 sample, 316 had taken part in both previous surveys. Figure 3 shows the number of participants by the date they began the survey, as well as when reminder emails were sent to previous participants.

### Recruitment

Similar recruitment methods were used for all three surveys.

Generation Scotland

Generation Scotland is a family health study of approximately 24,000 adults living in Scotland aged 18 to 99 years at recruitment from 2006 to 2011 ^22^. Participants who had children age 12 to 17 and for whom a working email address was known were sent an email prompting them to invite their children to take part in TeenCovidLife. Postal invitations were also sent to participants who had children in the appropriate age range, but for whom no email address was known.

SHINE network

SHINE is a network of over 500 schools that aims to bring together schools, policymakers, and academic researchers to conduct schools-based health and well-being research, and to support health improvement planning and implementation. Of the 514 schools in the SHINE network, 138 were secondary schools. The SHINE network helped promote the study to member schools, particularly for Survey 1.

Before the launch of Survey 1, the SHINE network announced the TeenCovidLife survey as the headline item in its May 2020 newsletter to all existing SHINE school members. The benefits of participation were outlined to schools, including TeenCovidLife’s incorporation of measures from the SHINE mental health survey, additional support from the SHINE team in promoting the study in school, and the offer of a school-level report. Additionally, one of the SHINE schools recorded a promotional video encouraging participation in the TeenCovidLife survey. This video was featured on the SHINE website and Twitter account.

Surveys 2 and 3 were likewise promoted to SHINE schools via the monthly newsletter and Twitter. However, school-level reports were not offered for these subsequent surveys.

General public

In addition to these recruitment routes, all three TeenCovidLife surveys were open to anyone age 12 to 17 living in Scotland. Both mainstream media and social media were used to advertise the study to the general public and encourage participation, as well as University of Edinburgh outreach programmes. Paid social media campaigns were run on Twitter, Instagram, and Facebook, and the surveys were also promoted through public engagement talks hosted by members of Generation Scotland.

Previous participants

In Surveys 2 and 3, participants who had taken part in a previous survey and provided a valid email address were re-contacted and invited to take part. Re-invited participants were sent a personalised link that gave them access to the survey and linked new responses to those from previous surveys.

### Procedure

A link to the study was included in emails and postal study invitations. A link to the study was also shared on social media and the Generation Scotland website. On arriving at the TeenCovidLife landing page, participants first read the volunteer information sheet. Participants also answered two questions to check they had read and understood the information sheet. Participants could not proceed to the main consent form until they answered both of these questions correctly. Next, participants completed the online consent form. Participants also gave consent to future re-contact from Generation Scotland. The consent form highlighted that they were not obliged to take part in future studies if they were re-contacted. Consent and information sheet text for each survey are available in the *Extended data* ^8^.

## Results

### TeenCovidLife Survey 1

**Figure 4.**
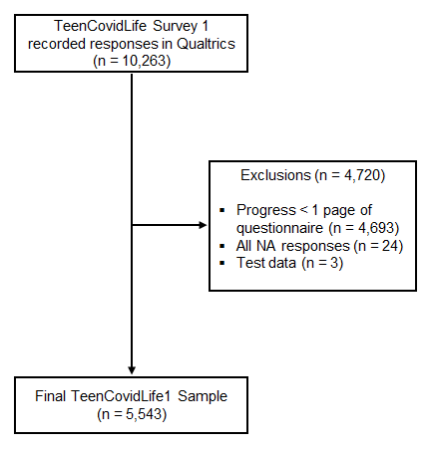
TeenCovidLife Survey 1 exclusions.

The data cleaning process is presented in Figure 4. A total of 10,263 participants accessed the survey during the recruitment stage. After data cleaning, 5,543 participants were included in the final sample. Respondents were retained as participants if they had a) completed and agreed to the consent form; b) progressed past the first page of the questionnaire, which contained only basic demographic information; and c) answered at least one of the questions. Two members of the research team conducted the data cleaning separately. Final records were compared, and any inconsistencies were investigated and resolved until both researchers had identified the same records for inclusion.

The time to complete the survey varied, as participants could save their data so far and complete the survey later. The median time taken to complete the survey was 21 minutes, with an interquartile range of 15 minutes.

**Figure 5.**
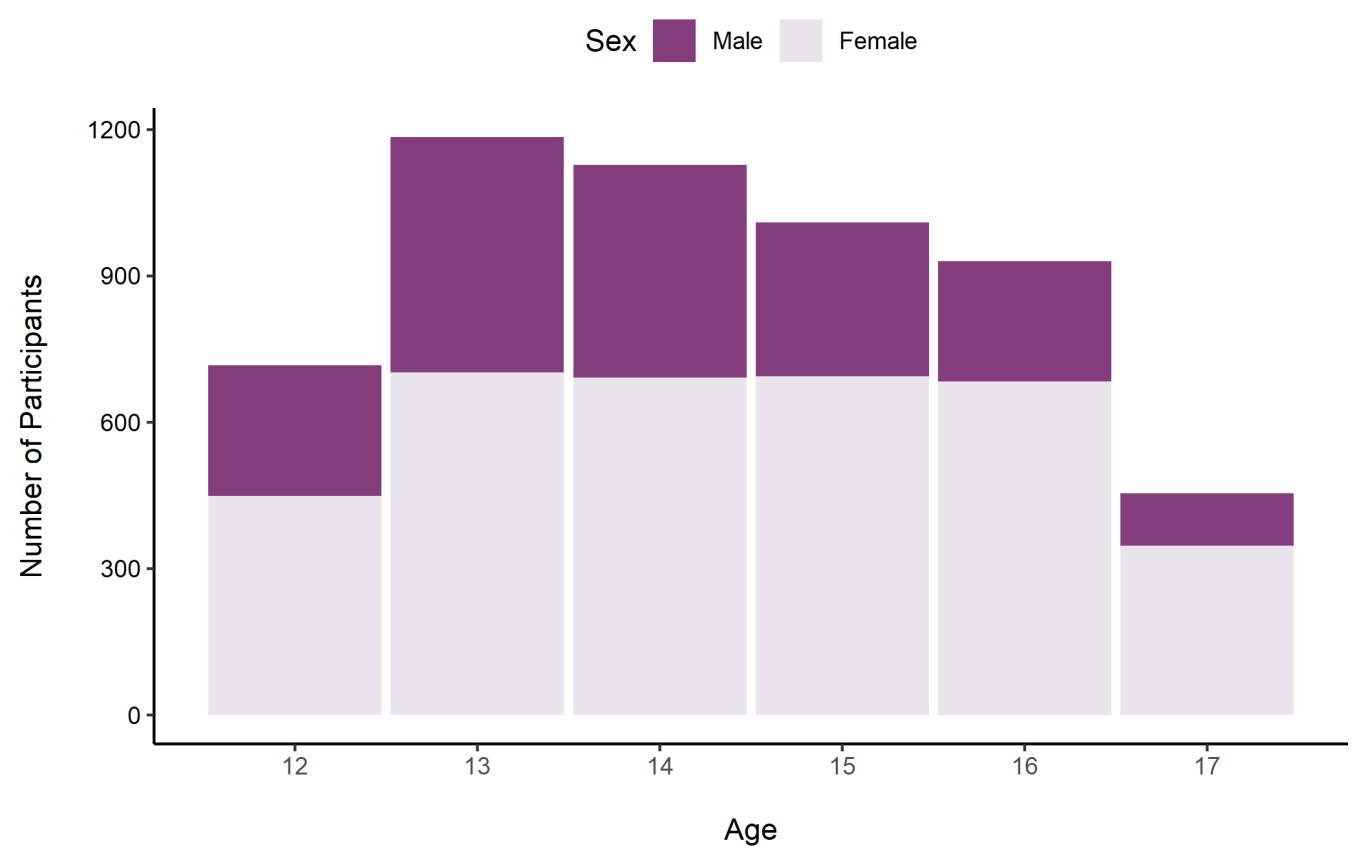
Number of TeenCovidLife Survey 1 participants by age and sex.

The sample was predominantly female (63.2%; 3,505), and there were slightly more participants in the 12 – 14 age group (55.5%; 3,074) than the 15 – 17 age group (43.6%; 2,415). Figure 5 shows the number of participants by age and sex.

Over half of the participants (2,933, 52.9%) were from urban areas, 12.5% (694) were from rural areas, and 24.2% (1,341) were from small towns. It is estimated that 17% of Scotland’s population lives in rural areas ^23^, indicating that rural participants may be slightly under-represented in this sample. No data was available on rural-urban classification for the remaining 10.4% (575) of participants.

The majority of the sample was white (84.4%, 4,678). This is expected as 2011 census data indicates 96.0% of Scotland identified as white ^24^. Almost half (44.3%; 2,456) came from schools with less than 10% pupils from deprived areas. Deprivation was assessed by examining the percentage of students at the participant’s school who lived in the most deprived quintile, based on the 2016 Scottish Index of Multiple Deprivation ^25^. Full demographic details of the sample are reported in Table 2.

**Table 2.** Demographic characteristics of TeenCovidLife Survey 1 Participants.

Sex (as registered at birth), n (%)	
Male	1,868 (33.7%)
Female	3,592 (64.8%)
Prefer not to answer	64 (1.2%)
Missing	19 (0.3%)
Gender Identity, n (%)	
Gender differs from sex	137 (2.5%)
Male	1,892 (33.4%)
Female	3,505 (63.2%)
Non-Binary or Other	70 (1.3%)
Prefer not to answer	57 (1.0%)
Missing	19 (0.3%)
Age, n (%)	
Mean Age (SD)	14.3 (1.5)
Age 12 – 14	3,074 (55.5%)
Age 15 – 17	2,415 (43.6%)
Ethnicity, n (%)	
White Scottish	4,135 (74.6%)
White Other	543 (9.8%)
Asian or Asian British	97 (1.7%)
Black or Black British	38 (0.7%)
Mixed	124 (2.3%)
Any other ethnicity	31 (0.6%)
Prefer not to say	72 (1.3%)
Missing	503 (9.1%)
Urban Rural Classification, n (%) ^1^	
Large urban areas	1,062 (19.2%)
Other urban areas	1,871 (33.8%)
Accessible small towns	720 (13.0%)
Remote small towns	621 (11.2%)
Accessible rural areas	586 (10.6%)
Remote rural areas	108 (1.9%)
Missing	575 (10.4%)
Deprivation, n (%) ^2^	
< 10%	2,456 (44.3%)
10 < 20%	391 (7.1%)
20 < 30%	531 (9.6%)
30 < 40%	327 (6.2%)
40% +	217 (3.9%)
Missing	1,621 (29.2%)
Other Factors, n (%)	
Has long-term medical condition	673 (12.1%)
Acts as a carer to household member	720 (13.0%)

^1^ Based on the Scottish Government Urban-Rural Classification 2016 ^2^ Based on percentage of students at participant’s school classified as deprived

Participants were from 287 different schools in all 32 local authority areas across Scotland. School data was not available for 10.0% (557) of participants. The local authority area with the highest number of participants was the Scottish Borders, representing 24.0% (1,329) of the sample. All Scottish Borders schools are members of the SHINE network.

Table 3 shows summary statistics for the commonly used psychological measures included in the study. Other summary statistics can be seen in the TeenCovidLife Survey 1 General Report ^26^, available on the Generation Scotland website.

**Table 3.** Summary statistics for commonly used psychometric measures in TeenCovidLife Survey 1.

Measure	*n*	Mean	*sd*
Adolescent Sleep-Wake Scale (10-item) [ASWS]			
Total	5,180	3.61	1.11
Falling Asleep & Reinitiating Sleep	5,184	4.15	1.34
Returning to Wakefulness	5,191	2.97	1.41
Going to Bed	5,221	3.15	1.37
Brief Resilience Scale [BRS]			
Total	5,292	3.13	0.76
Perceived Stress Scale (4-item) [PSS-4]			
Total	5,230	7.35	3.47
World Health Organisation Well-Being Index [WHO-5]			
Total	5,230	45.73	22.76
Social Emotional Health Survey [SEHS]			
Family Support	4,857	9.73	2.33
Peer Support	4,873	10.00	2.51
School Support	4,679	9.96	2.20
Optimism	4,936	7.94	2.57
Self-Efficacy	4,953	9.13	1.93

*Note.* *n* indicates number of participants who answer every question included in calculated scale

### TeenCovidLife Survey 2

**Figure 6.**
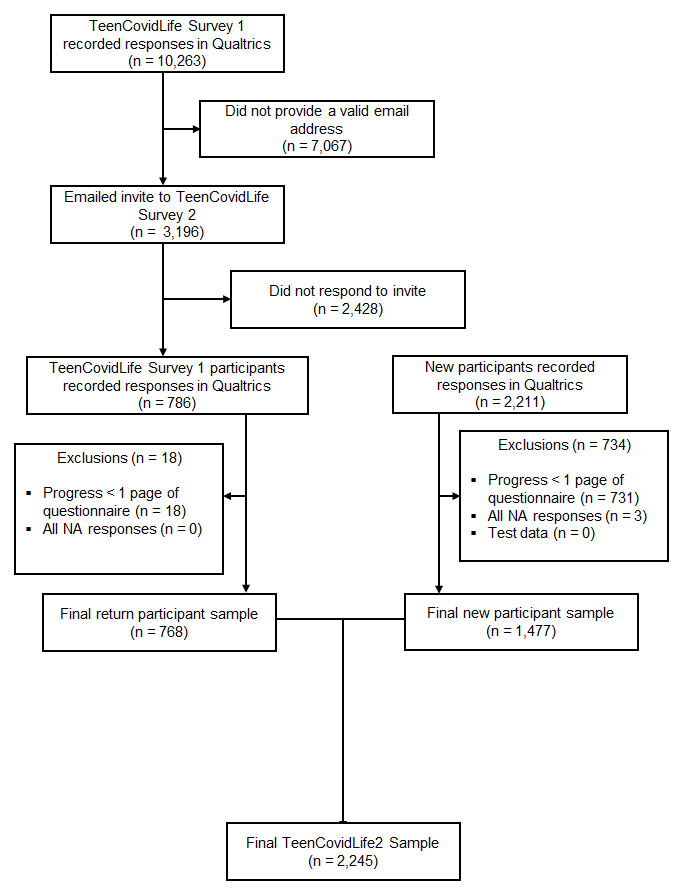
Flow chart of participants recruited for TeenCovidLife Survey.

During recruitment, 2,997 participants accessed the survey. Of these, 2,245 participants were included in the final dataset. From TeenCovidLife Survey 1, 3,196 previous participants were directly invited to take part. Of Survey 1 participants invited, 24.0% (768) responded and were included in the final sample. Data were cleaned in the same manner as in TeenCovidLife Survey 1. See Figure 6 for exclusions at each stage of the data cleaning.

Over a third (34.2%; 768) of the final sample had taken part in TeenCovidLife Survey 2, with an overall follow-up rate of 13.9% from Survey 1. As some older participants had birthdays between the first and second surveys, TeenCovidLife Survey 2 also includes data from 18-year-old participants.

As in Survey 1, participants could save their responses and return to the study later, meaning the time taken to complete the survey was highly variable. The median time taken to complete the survey was 18 minutes, with an interquartile range of 15 minutes.

**Figure 7.**
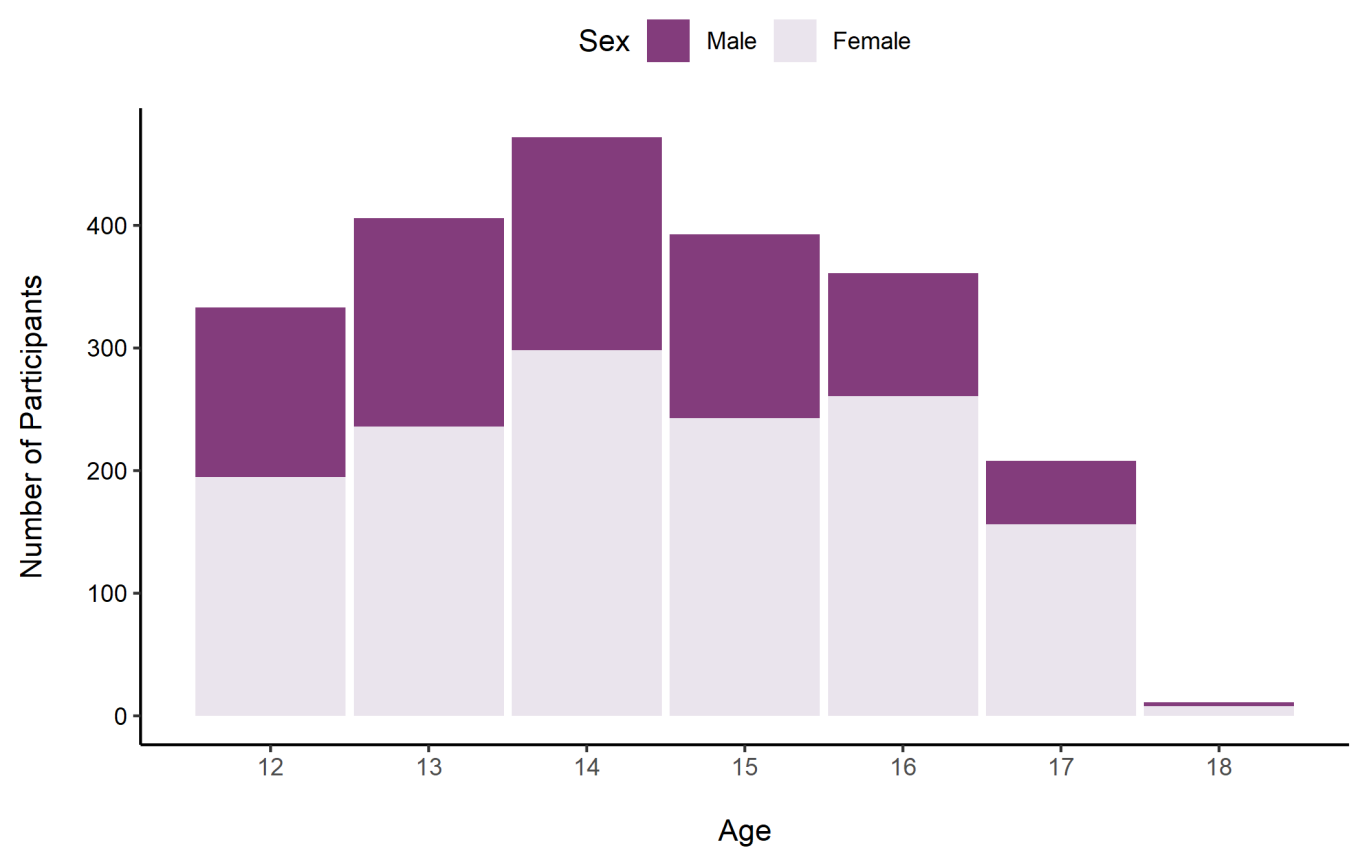
Number of TeenCovidLife Survey 2 participants by age and sex.

As in TeenCovidLife Survey 1, the sample was majority female (62.7%), and there were slightly more participants in the 12 – 14 age group (55.2%) than the 15 – 18 age group (43.8%). Figure 7 shows the sex ratio by age.

Almost half of the participants (46.1%, 1,029) were from urban areas, with 28.4% (635) from rural areas. As in Survey 1, the majority of the sample was white (82.8%, 1,847), and almost half (46.2%, 1,032) were from schools with 10% or fewer pupils living in deprived areas. Full demographic details of the sample can be seen in Table 4.

**Table 4.** Demographic characteristics of TeenCovidLife Survey 2 Participants.

Sex as registered at birth, n (%)	
Male	794 (35.4%)
Female	1,404 (62.5%)
Prefer not to answer/No response	47 (2.1%)
Gender Identity, n (%)	
Gender differs from sex	64 (2.9%)
Male	795 (35.4%)
Female	1,373 (61.2%)
Non-Binary or Other	34 (1.5%)
Prefer not to answer/No response	43 (1.9%)
Age, n (%)	
Mean Age (SD)	14.3 (1.6)
Age 12 – 14	1,239 (55.2%)
Age 15 – 18	981 (43.7%)
Ethnicity, n (%)	
White Scottish	1,651 (73.5%)
White Other	203 (9.0%)
Asian or Asian British	68 (3.0%)
Black or Black British	14 (0.6%)
Mixed	53 (2.4%)
Any other ethnicity	16 (0.7%)
Prefer not to say	26 (1.2%)
Missing	214 (9.5%)
Urban Rural Classification, n (%) ^1^	
Large urban areas	774 (34.5%)
Other urban areas	267 (11.9%)
Accessible small towns	200 (8.9%)
Remote small towns	165 (7.3%)
Accessible rural areas	629 (28.0%)
Remote rural areas	14 (0.6%)
Deprivation, n (%) ^2^	
< 10%	1,045 (46.5%)
10 < 20%	287 (12.8%)
20 < 30%	93 (4.1%)
30% +	87 (3.9%)
Missing	669 (29.8%)
Other Factors, n (%)	
Has long-term medical condition	267 (11.9%)
Acts as carer to household member	470 (20.9%)
Has an Autism Spectrum Condition (ASC)	80 (3.6%)
Has Attention-Deficit Hyperactivity Disorder (ADHD)	58 (2.6%)

^1^ Based on the Scottish Government Urban-Rural Classification 2016 ^2^ Based on percentage of students at participant’s school classified as deprived

Participants were from 166 different schools across Scotland over 31 local authority areas. School data was not available for 8.3% (186) participants. The most frequent local authority area was Falkirk, accounting for 24.5% (551) of the sample. This may relate to a SHINE school in the Falkirk area that showed a high response rate.

Table 5 shows summary statistics for the commonly used psychological measures included in the study. Other summary statistics can be seen in the TeenCovidLife Survey 2 General Report ^27^, as well as in the Exams Mini Report ^28^. Both are available for free download on the Generation Scotland website.

**Table 5.** Summary statistics for commonly used psychometric measures in TeenCovidLife Survey 2.

Measure	*n*	Mean	*sd*
Adolescent Sleep-Wake Scale (10-item) [ASWS]			
Total	1,956	3.60	1.08
Falling Asleep & Reinitiating Sleep	1,958	4.12	1.34
Returning to Wakefulness	1,958	2.69	1.35
Going to Bed	1,966	3.33	1.31
Brief Resilience Scale [BRS]			
Total	2,149	3.09	.75
Perceived Stress Scale (4-item) [PSS-4]			
Total	2,054	7.46	3.36
World Health Organisation Well-Being Index [WHO-5]			
Total	2,054	47.60	22.90
Social Emotional Health Survey [SEHS]			
Family Support	1,905	9.61	2.36
Peer Support	1,938	9.93	2.58
School Support	1,740	9.88	2.26
Optimism	1,805	7.79	2.68
Self-Efficacy	1,968	9.15	2.05

*Note.* *n* indicates number of participants who answer every question included in calculated scale

### TeenCovidLife Survey 3

**Figure 8.**
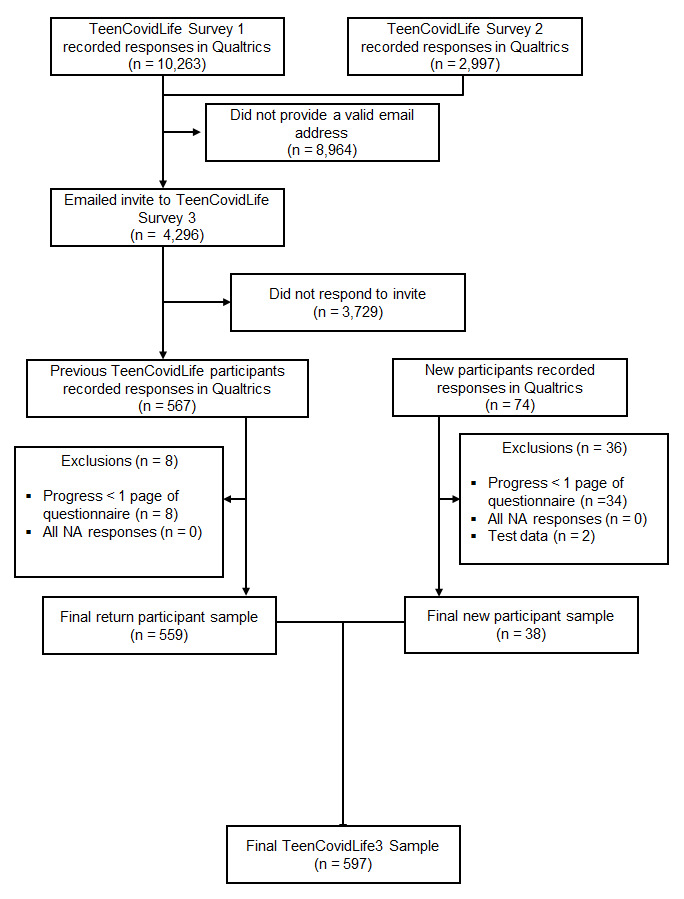
Flow chart of participants recruited for TeenCovidLife Survey 3.

A total of 641 participants accessed the survey during the recruitment stage. Of these, 597 participants completed the survey with a high enough rate of completion to be included in the final dataset. Data were cleaned in the same manner as in previous surveys. Figure 8 summarises the exclusions at each stage of the data cleaning.

**Figure 9.**
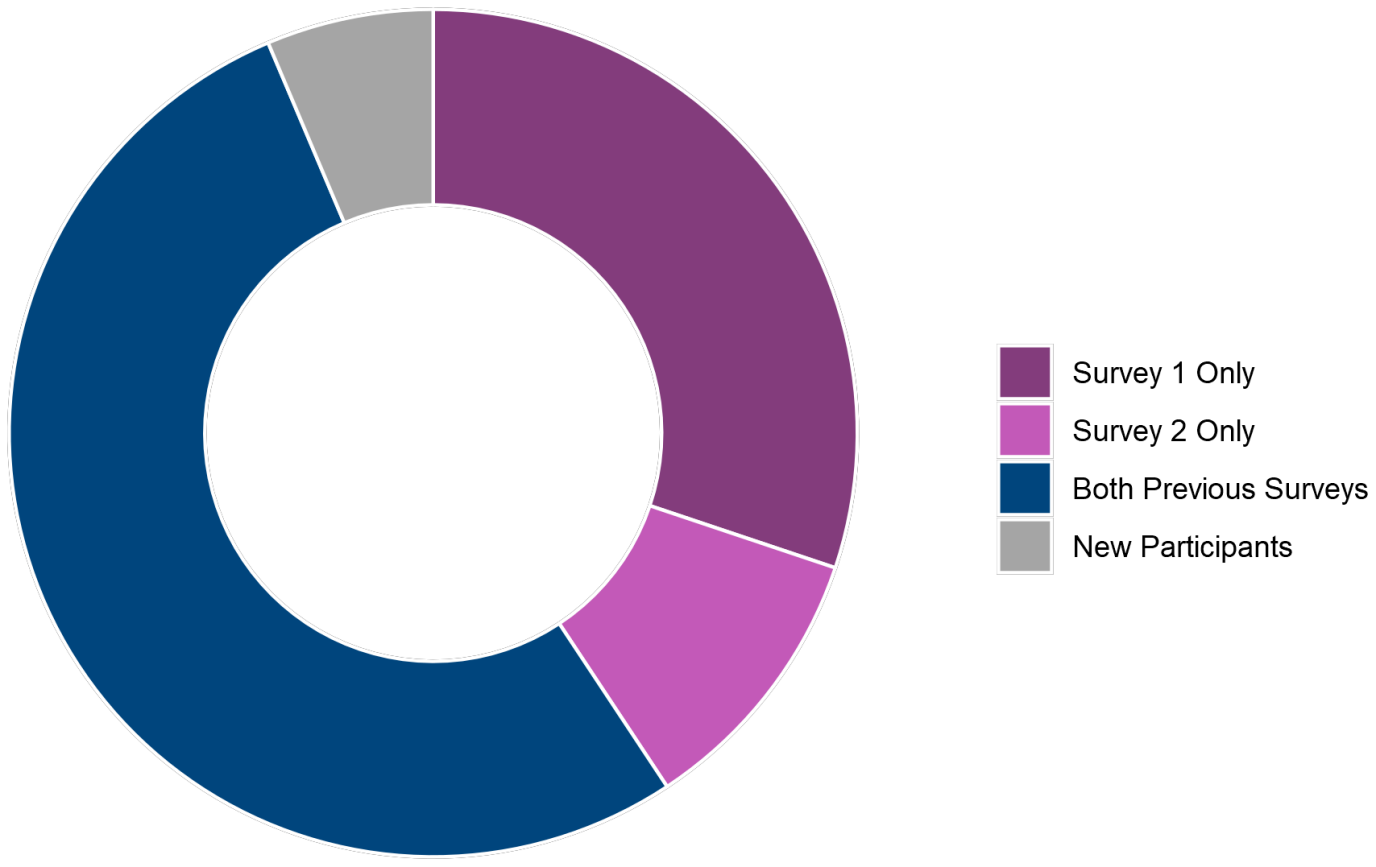
Doughnut plot of Survey 3 participants' participation in previous TeenCovidLife surveys.

The majority of participants (93.6%; 559) had taken part in at least one previous TeenCovidLife Survey. Figure 9 shows how many Survey 3 participants had taken part in previous TeenCovidLife surveys. Over half (52.9%; 316) had taken part in both Survey 1 and Survey 2., 30.2% (180) had taken part in only Survey 1, and 10.6% (63) had taken part in only Survey 2.

As in previous surveys, participants could save their responses and return to the study at a later date, meaning the time taken to complete the survey was highly variable. The median time taken to complete the survey was 11 minutes, with an interquartile range of seven minutes.

**Figure 10.**
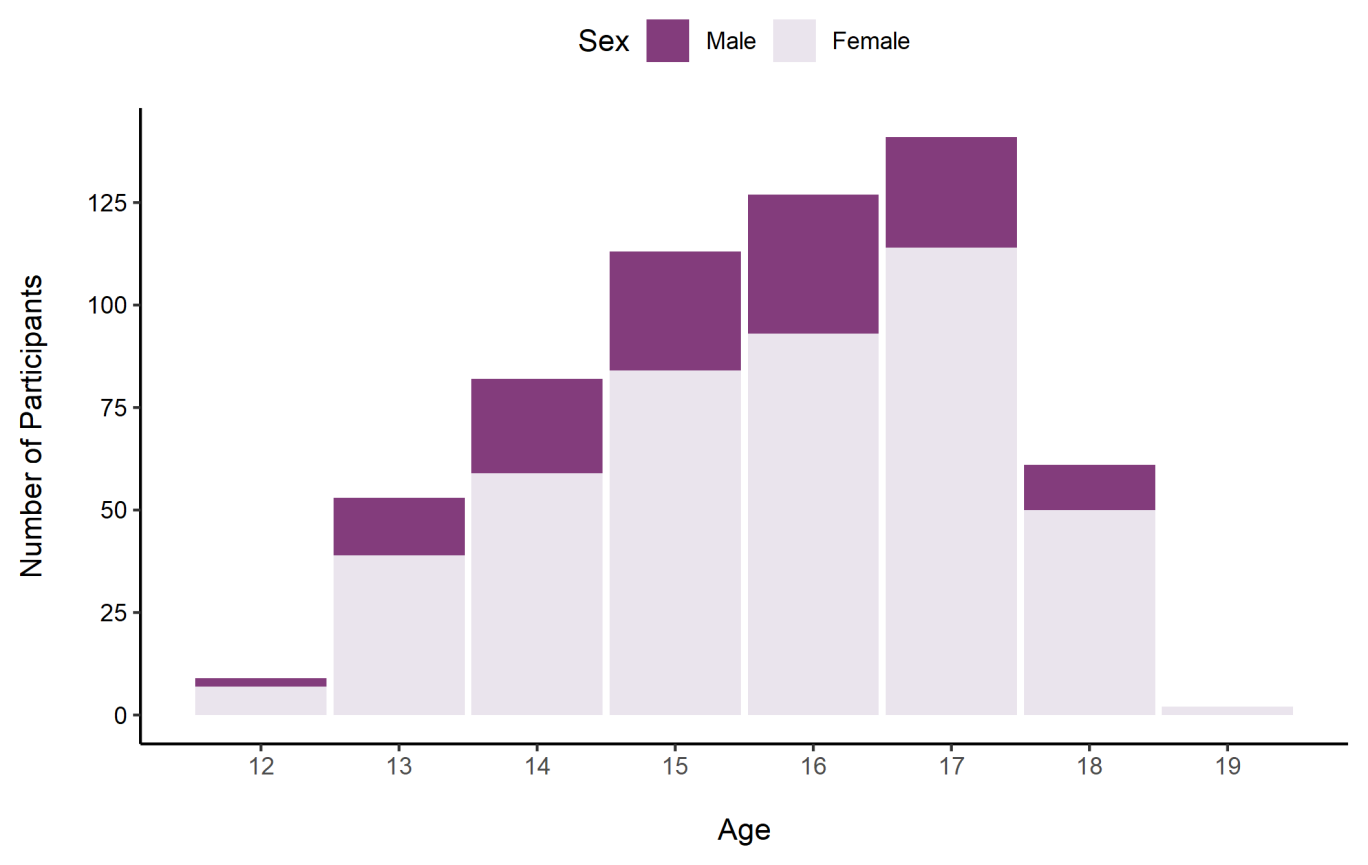
Number of TeenCovidLife Survey 3 participants by age and sex.

As in the previous surveys, the sample was majority female (72.7%). As some returning participants may have turned 19 since the first survey, the sample included participants up to age 19. The majority of participants were in the 15 – 19 age group (75.2%). Figure 10 shows the number of participants in each age band by sex.

Over half of the participants (55.4%, 331) were from urban areas, with 13.2% (79) from rural areas. As in previous surveys, the majority of the sample was white (86.8%, 518), and almost half (45.7%, 273) were from schools with 10% or fewer pupils living in deprived areas. Full demographic details of the sample can be seen in Table 6.

**Table 6.** Demographic characteristics of TeenCovidLife Survey 3 Participants.

Sex as registered at birth, n (%)	
Male	140 (23.5%)
Female	448 (75.0%)
Prefer not to answer/No response	< 10
Gender Identity, n (%)	
Gender differs from sex	22 (3.7%)
Male	143 (24.0%)
Female	434 (72.7%)
Non-Binary or Other	11 (1.8%)
Prefer not to answer/No response	< 10
Age, n (%)	
Mean Age (SD)	15.64 (1.54)
Age 12 – 14	148 (25.8%)
Age 15 – 19	449 (75.2%)
Ethnicity, n (%)	
White Scottish	472 (79.1%)
White Other	46 (7.7%)
Non-White Ethnic Minority	32 (5.4%)
Prefer not to say/No response	47 (7.9%)
Urban Rural Classification, n (%) ^1^	
Large urban areas	194 (32.5%)
Other urban areas	137 (22.9%)
Accessible small towns	68 (11.4%)
Remote small towns	66 (11.1%)
Accessible rural areas	69 (11.5%)
Remote rural areas	10 (1.7%)
Deprivation, n (%) ^2^	
<10%	273 (45.7%)
10 < 20%	62 (10.4%)
20 < 30%	48 (8.0%)
30% +	52 (8.7%)
Missing	122 (20.4%)
Other Factors, n (%)	
Has long-term medical condition	103 (17.3%)
Acts as carer to household member	67 (11.2%)
Has an Autism Spectrum Condition (ASC)	22 (3.7%)
Has Attention-Deficit Hyperactivity Disorder (ADHD)	11 (1.8%)

^1^ Based on the Scottish Government Urban-Rural Classification 2016 ^2^ Based on percentage of students at participant’s school classified as deprived

Participants were from 146 different schools across 31 local authority areas in Scotland. As in Survey 1, the most frequent local authority area was the Scottish Borders, accounting for 16.1% (96) of the sample.

Table 7 shows summary statistics for the commonly used psychological measures included in the study. As the majority of participants took part in previous surveys, only measures that had been used again in Survey 3 are included here.

**Table 7.** Summary statistics for commonly used psychometric measures in TeenCovidLife Survey 3.

Measure	*n*	Mean	*sd*
Adolescent Sleep-Wake Scale (10-item) [ASWS]			
Total	493	3.43	1.10
Falling Asleep & Reinitiating Sleep	496	3.94	1.34
Returning to Wakefulness	494	2.49	1.31
Going to Bed	495	3.21	1.41
Perceived Stress Scale (4-item) [PSS-4]			
Total	493	3.43	1.10
World Health Organisation Well-Being Index [WHO-5]			
Total	548	39.57	20.82
Social Emotional Health Survey [SEHS]			
School Support	427	10.02	2.20
Optimism	480	6.97	2.64

*Note.* *n* indicates number of participants who answer every question included in calculated scale

### Full participation subsample

A subsample of 316 participants took part in all three surveys, indicating a 5.7% complete follow-up rate from Survey 1. Table 8 shows the demographic details of this sample. The subsample was heavily skewed towards female participants, with only 21.2% being male. The majority of participants were white (94.0%; 297), and over half went to school in urban areas (59.8%; 189). The mean age at Survey 1 was 14.94 (SD = 1.48). At Survey 3, this was 15.92 (SD = 1.50).

**Table 8.** Demographic characteristics of TeenCovidLife participants who took part in all three surveys.

Sex as registered at birth, n (%)	
Male	64 (20.2%)
Female	249 (78.8%)
Prefer not to answer/No answer	< 10
Gender Identity, n (%)	
Gender differs from sex	12 (3.8%)
Male	67 (21.2%)
Female	239 (75.6%)
Non-Binary or Other	< 10
Prefer not to answer/No answer	< 10
Ethnicity, n (%)	
White Scottish	271 (85.8%)
White Other	26 (8.2%)
Non-white Ethnic Minority	18 (5.7%)
Prefer not to say/No answer	< 10
Urban Rural Classification, n (%) ^1^	
Urban areas	189 (59.8%)
Small towns	84 (26.6%)
Rural areas	32 (10.1%)
Deprivation, n (%) ^2^	
< 10%	159 (50.3%)
10 < 20%	31 (9.8%)
20 < 30%	26 (8.2%)
30% +	27 (8.5%)
Missing	73 (23.1%)
Other Factors, n (%)	
Has long-term medical condition	50 (15.8%)
Acts as carer to household member	30 (9.5%)
Has an Autism Spectrum Condition (ASC)	15 (4.7%)
Has Attention-Deficit Hyperactivity Disorder (ADHD)	< 10
Psychological Outcomes, mean (SD)	
Brief Resilience Scale (BRS)	3.12 (.81)
Family Support	9.36 (2.28)
Peer Support	9.54 (2.84)
Self-Efficacy	9.18 (2.06)

^1^ Based on the Scottish Government Urban-Rural Classification 2016 ^2^ Based on percentage of students at participant’s school classified as deprived

## Strengths and limitations

### Strengths

The core strengths of this dataset are that it is timely, rich, and longitudinal – few other cohorts have assessed the impact of the COVID-19 pandemic in such a large sample of adolescents. The COVID-19 pandemic has led to disruptions to long-term education, such as through school closures and cancelled exams. These disruptions may have long-term effects on health, well-being and success of young people, warranting study for years to come.

TeenCovidLife was designed in cooperation with the schools-based health behavioural research study SHINE. SHINE also forms part of the wider Health Behaviours in School-Aged Children study ^9, 29^. TeenCovidLife uses many of the same measures and questions as in both HBSC studies, as well as SHINE projects such as the SHINE networks pupil mental health and wellbeing survey. This harmonisation facilitates cross-cohort comparisons. Moreover, SHINE’s expertise ensured TeenCovidLife asked questions relevant and meaningful to young people.

Finally, the surveys were implemented at key time points – during the first period of school closures in the UK, when schools were beginning to open again and lockdown measures were easing, and finally a year after the first lockdown, following the second national lockdown. This allows researchers to assess the impact of school closures, as well as the long-term effects of the pandemic on young people over time.

### Limitations

The study was restricted to those with internet use, due to the need to adhere to COVID-19 mitigation measures. As such, those from rural communities or lower socioeconomic backgrounds with less stable internet access may be under-represented. The sample was also self-selected, meaning more altruistic or conscientious young people may have been more likely to take part.

As can be seen in the demographics, the dataset is not representative of the general adolescent population, with female participants being over-represented. Additionally, over 80% of the participants in all three surveys were white. While 2011 census data indicates Scotland’s population is 96.0% white ^24^, making this relatively expected, the low number of ethnic minority participants limits the analyses that can be conducted on ethnicity.

There also seemed to be an over-representation of young people with caring responsibilities. Between 12 to 21% of TeenCovidLife participants cared for a member of their household, while 2011 Scottish census data suggests only 3% of young people age 4 to 24 identify as carers ^30^. This may reflect a misunderstanding of the survey item, particularly if young people were taking more responsibility for younger siblings during the lockdowns, or some bias in the recruitment process. It is also possible that those with caring responsibilities were more likely to be interested in the project.

Furthermore, there were relatively low follow-up rates. Only 5.7% who participated in Survey 1 also took part in Survey 2 and 3. However, although at early stages participants were informed that they may be contacted for future surveys, this was not a defined goal of the study from the outset. Survey 1’s recruitment was also considerably larger as schools were mostly closed, with young people’s studies relatively disrupted or often suspended. As such, participants had more time to take part. Moreover, the pandemic was an even more salient topic at this early stage. By comparison, Survey 3 was conducted when schools were open and most adolescents were in a very busy school assessment and exam period, and the pandemic had been on-going for over a year, meaning it was relatively less salient.

The impact of the SHINE network’s active promotion of TeenCovidLife Survey 1 may have also significantly contributed to the difference in uptake. The SHINE Network Manager, a former Deputy Headteacher, was able to advise schools accordingly to promote a whole school approach to data collection, appropriate during remote learning. While the poor retention remains a limitation, the sub-sample of participants involved at all three waves (n = 316) may still be useful for analysis.

Finally, most participants did not fully answer every single question. Due to ethical reasons, participants were permitted to skip questions that they were uncomfortable with or did not wish to answer. Consequently, there is missing data and incomplete items; see data dictionaries under *Extended data* for the completion rate for each question.

## Ethical considerations

The TeenCovidLife study was reviewed and given a favourable opinion by the East of Scotland Research Ethics Committee (Reference: 20/ES/0021 AM03).

## Conclusions

The data obtained through the TeenCovidLife project aimed to capture the impact of the COVID-19 pandemic on adolescents in Scotland. Three datasets were collected at three key time points for young people, assessing the emotional impact of both the pandemic and the national lockdowns on health, well-being, and education. A subsample of 316 participants took part in all three waves of data collection, allowing for analysis of change over time. This dataset is a valuable resource for researchers, and is available through the established data access procedure from Generation Scotland.

## Data availability

### Underlying data

Non-identifiable data from the TeenCovidLife surveys are available to researchers in the UK and internationally through authorised access. Researchers who wish to use the TeenCovidLife data can apply for access using the standard Generation Scotland application process. More information about the process can be found on the Generation Scotland website ( www.generationscotland.org).

### Extended data

Zenodo: Extended data for "TeenCovidLife: A resource to understand the impact of the Covid-19 pandemic on adolescents in Scotland", https://doi.org/10.5281/zenodo.5526056 ^8^

This project contains the following extended data:

- 2020-09-18 TeenCovidLife Survey1 Data Dictionary.xlsx
- 2020-11-26 TeenCovidLife Survey2 Data Dictionary.xlsx
- 2021-07-26 TeenCovidLife Survey3 Data Dictionary.xlsx
- 2021-09-22 TeenCovidLife 2 VIS and Consent.docx
- 2021-09-22 TeenCovidLife 3 VIS and Consent.docx
- 2021-09-22 TeenCovidLife1 Questionnaire.docx
- 2021-09-22 TeenCovidLife1 VIS and Consent.docx
- 2021-09-22 TeenCovidLife2 Questionnaire NEW PARTICIPANTS.docx
- 2021-09-22 TeenCovidLife2 Questionnaire REPEAT PARTICIPANTS.docx
- 2021-09-22 TeenCovidLife3 Questionnaire NEW.docx
- 2021-09-22 TeenCovidLife3 Questionnaire REPEAT.docx
- 2021-09-22_STROBE_checklist_TeenCovidLife_DataNote_v1.0.docx
- CovidLife_Access_Request_Form_V3.1_March_2021.docx
- Generation_Scotland_Access_Request_Form_V1.2_March_2021.docx

Data are available under the terms of the Creative Commons Attribution 4.0 International license (CC-BY 4.0).
