# Supplementary material for: TeenCovidLife: a resource to understand the impact of the COVID-19 pandemic on adolescents in Scotland
Source: Wellcome Open Res. 2022 May 24;6:277. Originally published 2021 Oct 18. [Version 2] doi: 10.12688/wellcomeopenres.17252.2 (PMC9360910; doi:10.12688/wellcomeopenres.17252.2)
Supplement: Peer review history

# Peer review: Reviewer response for version 2

**DOI:** 10.21956/wellcomeopenres.19834.r51268

**Published:** 2022-08-08

**Authors:** Cerisse Gunasinghe

**Affiliations:** 1 Department of Psychological Medicine, Institute of Psychiatry, Psychology and Neuroscience, King's College London, London, UK

This is an invaluable programme of research with important findings which have implications for further research and intervention.

- In the version reviewed, there is a spelling error in the second paragraph, last sentence of the section 'Questionnaire Development'.
- It might be useful to add how the research team might have been able to improve recruitment of underrepresented groups, particularly those from deprived areas as well as racial and ethnic minority young people who are at equal or even higher risk of health inequalities.
- Were parental consent and assent required for those under 16 years, if so, some clarity here might be useful to readers. 

Are sufficient details of methods and materials provided to allow replication by others?

Yes

Is the rationale for creating the dataset(s) clearly described?

Yes

Are the datasets clearly presented in a useable and accessible format?

Yes

Are the protocols appropriate and is the work technically sound?

Yes

Reviewer Expertise:

Mental Health. Health inequalities. Young people's health and social welfare. Interface with physical health. Health service use.

I confirm that I have read this submission and believe that I have an appropriate level of expertise to confirm that it is of an acceptable scientific standard.

# Peer review: Reviewer response for version 2

**DOI:** 10.21956/wellcomeopenres.19834.r50718

**Published:** 2022-06-10

**Authors:** Gemma Sicouri

**Affiliations:** 1 Black Dog Institute, University of New South Wales, Sydney, Sydney, NSW, Australia

I have reviewed the recent revisions and approve the paper in its current form.

Are sufficient details of methods and materials provided to allow replication by others?

Yes

Is the rationale for creating the dataset(s) clearly described?

Partly

Are the datasets clearly presented in a useable and accessible format?

Partly

Are the protocols appropriate and is the work technically sound?

Yes

Reviewer Expertise:

Youth mental health, youth mental health during COVID-19

I confirm that I have read this submission and believe that I have an appropriate level of expertise to confirm that it is of an acceptable scientific standard.

# Peer review: Reviewer response for version 1

**DOI:** 10.21956/wellcomeopenres.19068.r48975

**Published:** 2022-03-22

**Authors:** Gemma Sicouri

**Affiliations:** 1 Black Dog Institute, University of New South Wales, Sydney, Sydney, NSW, Australia

Thank you for the opportunity to review this paper providing information on three surveys conducted on adolescents during the COVID-19 pandemic in Scotland.

 The paper is well written, conceptualised, and the data collection procedures are well described. TeenCovidLife has the potential to be a useful resource for future researchers in tracking youth mental health and wellbeing during the COVID-19 pandemic in Scotland. My comments pertain to improving the overall impact and relevance of the paper. Providing further information on the representativeness of the sample needs to be addressed before recommending it for acceptance without reservations.

1. The rationale for the study in the introduction could be improved and updated with more recent research on adolescent wellbeing during COVID-19. Examples below:
  - (a) The authors reference the parent study to TeenCovidLife as indicating that the pandemic may have particularly affected young people (Fawns-Ritchie *et al*., 2021). Yet this is a preprint paper and not peer-reviewed. It would be recommended for the authors to reference a published study in addition to this study to support this point (e.g., Racine *et al*., 2021 ^1^ ).
  - (b) There have been a number of studies published as part of the Co-SPACE stream of research in the UK. This might provide a nice reference for how the mental health of young people is faring in the UK ( https://www.psy.ox.ac.uk/research/topic-research-group/supporting-parents-adolescents-and-children-during-epidemics).
  - (c) Given other research, the authors could highlight how this survey captures the experience of adolescents in Scotland in particular, and perhaps focus on measures that were relevant for tracking resilience as well as general wellbeing measures.
2. I am not completely clear on what the primary purpose of the paper is. Was it to describe a resource that collected survey data during the pandemic (and descriptives of that population), or were the authors aiming to analyse and describe the results of the data? It seems that the former is the aim that was fulfilled as there is no discussion on what the results showed (in comparison to each other or the overall pattern of results), in which case this needs to be made clearer in the introduction and aims section.
3. I am wondering if the authors could combine some of the information that they have detailed in individual tables for each survey time point into one table with a column for each time point. This makes it easy to compare across surveys and reduces the number of figures/tables needed. For example, could the demographic characteristics for each of the surveys (T1, T2, T3) be combined into one table with different columns for each time point?
4. It would be recommended for the authors to provide data on the representativeness of the sample obtained. The authors could include a column in the combined table with this information (e.g., % male in this age group) from population studies. This would be very helpful for users of the dataset to understand the representativeness of the sample (rather than some references to aspects of representativeness in the text).
5. Did the authors use any methods for dealing with missing data on individual items in the psychometric measures used in TeenCovidLife? For example, mean imputation? Please detail.
6. Minor point: are the authors confident that the sample was based on living in Scotland? Was there a question pertaining to this? Given the questionnaires were online, it is possible that participants from outside Scotland completed the measures.

Are sufficient details of methods and materials provided to allow replication by others?

Yes

Is the rationale for creating the dataset(s) clearly described?

Partly

Are the datasets clearly presented in a useable and accessible format?

Partly

Are the protocols appropriate and is the work technically sound?

Yes

Reviewer Expertise:

Youth mental health, youth mental health during COVID-19

I confirm that I have read this submission and believe that I have an appropriate level of expertise to confirm that it is of an acceptable scientific standard, however I have significant reservations, as outlined above.

## References

[1] : Global Prevalence of Depressive and Anxiety Symptoms in Children and Adolescents During COVID-19. *JAMA Pediatrics* .2021;175(11) : 10.1001/jamapediatrics.2021.2482 10.1001/jamapediatrics.2021.2482 PMC835357634369987

# Response

**Published:** 2022-05-16

**Authors:** Charlotte Huggins

**Affiliations:** University of Edinburgh, UK

Thank you very much to the reviewer for their kind and detailed response. We have incorporated their feedback and feel this has considerably strengthened the paper. Please find responses to more specific points below.

- *The rationale for the study in the introduction could be improved and updated with more recent research on adolescent wellbeing during COVID-19. Examples below:(a) The authors reference the parent study to TeenCovidLife as indicating that the pandemic may have particularly affected young people (Fawns-Ritchie et al., 2021). Yet this is a preprint paper and not peer-reviewed. It would be recommended for the authors to reference a published study in addition to this study to support this point (e.g., Racine et al., 20211).*
- **Response: **Thank you for this suggestion. We agree it would strengthen our position to cite a peer-reviewed study outside of the CovidLife family. This citation has been added to the introduction, see line 195 - 197, or below: “A meta-analysis of the prevalence of depression and anxiety among young people throughout the pandemic indicates that prevalence has increased and remains high [6].” 
- *(b) There have been a number of studies published as part of the Co-SPACE stream of research in the UK. This might provide a nice reference for how the mental health of young people is faring in the UK ( https://www.psy.ox.ac.uk/research/topic-research-group/supporting-parents-adolescents-and-children-during-epidemics). (c) Given other research, the authors could highlight how this survey captures the experience of adolescents in Scotland in particular, and perhaps focus on measures that were relevant for tracking resilience as well as general wellbeing measures.*
- **Response: **Thank you for pointing us towards this valuable resource and for this suggestion, a small line has been added to the introduction, see lines 205 - 208 or below: “This complements existing work such as the Co-SPACE stream of resources [8] by capturing the experiences of young people in Scotland in particular, using multiple measures to capture resilience and general well-being.”
- *I am not completely clear on what the primary purpose of the paper is. Was it to describe a resource that collected survey data during the pandemic (and descriptives of that population), or were the authors aiming to analyse and describe the results of the data? It seems that the former is the aim that was fulfilled as there is no discussion on what the results showed (in comparison to each other or the overall pattern of results), in which case this needs to be made clearer in the introduction and aims section.*
- **Response: **Thank you for your feedback.  This paper is intended as a data note, and as such is meant to describe the resource and how the data was collected. No analysis of interpretation of the results is intended here. A sentence stressing the primary purpose of the paper has been added to the introduction section, see lines 215 - 218, as follows: “This paper is a data note, and as such is intended to describe the TeenCovidLife data, as well as how it was collected, in order to act as reference for future researchers. Analysis and interpretation of the data and its potential implications for health and policy is beyond the scope of the current paper.”
- *I am wondering if the authors could combine some of the information that they have detailed in individual tables for each survey time point into one table with a column for each time point. This makes it easy to compare across surveys and reduces the number of figures/tables needed. For example, could the demographic characteristics for each of the surveys (T1, T2, T3) be combined into one table with different columns for each time point? It would be recommended for the authors to provide data on the representativeness of the sample obtained. The authors could include a column in the combined table with this information (e.g., % male in this age group) from population studies. This would be very helpful for users of the dataset to understand the representativeness of the sample (rather than some references to aspects of representativeness in the text).*
- **Response:** Thank you for this useful feedback. We have collapsed all the tables for each survey timepoint into one table, see Table 2, and included population estimates where data is reasonably available.
- *Did the authors use any methods for dealing with missing data on individual items in the psychometric measures used in TeenCovidLife? For example, mean imputation? Please detail.*
- **Response:** No missing data imputation was conducted, as appropriate ways to handle missing data may vary depending on the purpose of analysis. As such, we concluded that missing data will be left as-is, so researchers may make appropriate decisions for their analysis in future. Sum scores are available only for those with complete psychometric measures.
- *Minor point: are the authors confident that the sample was based on living in Scotland? Was there a question pertaining to this? Given the questionnaires were online, it is possible that participants from outside Scotland completed the measures.*
- **Response: **In the consent, participants confirmed that they were living in Scotland, please see Extended Data.
